# Emerging Use of Gene Expression Microarrays in Plant Physiology

**DOI:** 10.1002/cfg.277

**Published:** 2003-04

**Authors:** Stan D. Wullschleger, Stephen P. Difazio

**Affiliations:** Environmental Sciences Division Oak Ridge National Laboratory Oak Ridge TN TN 37831-6422 USA

## Abstract

Microarrays have become an important technology for the global analysis of gene expression in humans, animals, plants, and microbes. Implemented in the context of a well-designed experiment, cDNA and oligonucleotide arrays can provide highthroughput, simultaneous analysis of transcript abundance for hundreds, if not thousands, of genes. However, despite widespread acceptance, the use of microarrays as a tool to better understand processes of interest to the plant physiologist is still being explored. To help illustrate current uses of microarrays in the plant sciences, several case studies that we believe demonstrate the emerging application of gene expression arrays in plant physiology were selected from among the many posters and presentations at the 2003 Plant and Animal Genome XI Conference. Based on this survey, microarrays are being used to assess gene expression in plants exposed to the experimental manipulation of air temperature, soil water content and aluminium concentration in the root zone. Analysis often includes characterizing transcript profiles for multiple post-treatment sampling periods and categorizing genes with common patterns of response using hierarchical clustering techniques. In addition, microarrays are also providing insights into developmental changes in gene expression associated with fibre and root elongation in cotton and maize, respectively. Technical and analytical limitations of microarrays are discussed and projects attempting to advance areas of microarray design and data analysis are highlighted. Finally, although much work remains, we conclude that microarrays are a valuable tool for the plant physiologist interested in the characterization and identification of individual genes and gene families with potential application in the fields of agriculture, horticulture and forestry.


**Comparative and Functional Genomics**
*Comp Funct Genom* 2003; **4**: 216–224.
Published online 1 April 2003 in Wiley InterScience (www.interscience.wiley.com). **DOI:** 10.1002/cfg.277

**Conference Review**

### Emerging use of gene expression microarrays in plant physiology

Stan D. Wullschleger* and Stephen P. Difazio
*Environmental Sciences Division, Oak Ridge National Laboratory, Oak Ridge, TN 37831-6422, USA*

*Correspondence to:
Stan D. Wullschleger,
Environmental Sciences Division,
Oak Ridge National Laboratory,
Oak Ridge, Tennessee,
37831-6422, USA.
E-mail: wullschlegsd@ornl.gov*

### Abstract

**Microarrays have become an important technology for the global analysis of gene expression in humans, animals, plants, and microbes. Implemented in the context of a well-designed experiment, cDNA and oligonucleotide arrays can provide high-throughput, simultaneous analysis of transcript abundance for hundreds, if not thousands, of genes. However, despite widespread acceptance, the use of microarrays as a tool to better understand processes of interest to the plant physiologist is still being explored. To help illustrate current uses of microarrays in the plant sciences, several case studies that we believe demonstrate the emerging application of gene expression arrays in plant physiology were selected from among the many posters and presentations at the 2003 Plant and Animal Genome XI Conference. Based on this survey, microarrays are being used to assess gene expression in plants exposed to the experimental manipulation of air temperature, soil water content and aluminium concentration in the root zone. Analysis often includes characterizing transcript profiles for multiple post-treatment sampling periods and categorizing genes with common patterns of response using hierarchical clustering techniques. In addition, microarrays are also providing insights into developmental changes in gene expression associated with fibre and root elongation in cotton and maize, respectively. Technical and analytical limitations of microarrays are discussed and projects attempting to advance areas of microarray design and data analysis are highlighted. Finally, although much work remains, we conclude that microarrays are a valuable tool for the plant physiologist interested in the characterization and identification of individual genes and gene families with potential application in the fields of agriculture, horticulture and forestry.** **Copyright © 2003 John Wiley & Sons, Ltd.**

Received: 5 February 2003
Revised: 5 February 2003
Accepted: 6 February 2003

**Keywords: cDNA; expression profile; genomics; hybridization; oligonucleotide; plant physiology; stress**

### Introduction

Since their introduction in the mid-1990s, cDNA and oligonucleotide microarrays have been embraced by the biological community for the global analysis of gene expression in prokaryotic and eukaryotic organisms [24]. DNA microarrays represent an unprecedented analytical tool for transcript profiling. Implemented in the context of a well-designed experiment, microarrays provide high-throughput, simultaneous analysis of mRNA for hundreds, if not thousands, of genes [1,19]. Such comprehensive analysis provides the opportunity to explore molecular mechanisms that underlie a variety of plant physiological processes, and therein cDNA and oligonucleotide arrays can be used to gain a direct link to gene function [23]. Thus, by correlating changes in gene expression with changes in physiology, it should be possible to derive insight into a broad range of biological processes. Microarrays have already been used to characterize genes involved in the regulation

Copyright © 2003 John Wiley & Sons, Ltd.

of circadian rhythms, plant defence mechanisms, oxidative stress responses, fruit ripening, phytochrome signalling, seed development and nitrate assimilation [1].

Despite their impressive and rapidly growing acceptance in the biological sciences, the use, analysis and interpretation of data from microarrays in plant physiology is still developing. In this review, several research projects that we believe demonstrate the emerging application of gene expression microarrays in the field of plant physiology were selected from among the many posters and presentations at the Plant and Animal Genome XI Conference. The case studies highlighted here represent a survey of research being conducted in which microarrays serve as the central technological platform. These studies demonstrate how DNA microarrays are enabling the plant physiologist to ask questions that were not possible just a few years ago and, by example, illustrate how the field of plant physiology is being expanded by the availability of this emerging technology.

### Microarray technology

Most microarrays used in the biological sciences today can be divided into two groups: complementary DNA (cDNA) and oligonucleotide microarrays [25]. This division refers to characteristics of the probes, the individual pieces of gene-specific DNA that are immobilized on the array surface. cDNA probes are usually products of the polymerase chain reaction (PCR) generated from cDNA libraries or genomic DNA, and are typically in excess of 150 nucleotides in length. On the other hand, synthetic oligonucleotides have a maximum length of around 80 nucleotides, thus conferring greater specificity among members of gene families [1,15,18].

Array fabrication involves either spotting of presynthesized probes using highly precise robots, or *in situ* synthesis on glass slides. High-density spotted microarrays can contain up to 40 000 probes on a conventional microscope slide. In contrast, arrays consisting of gene-specific oligonucleotides can be synthesized directly onto a solid surface by either photolithography or ink-jet technology [23]. Since sequence information by itself is sufficient to generate the DNA probes to be arrayed, probes can be designed to represent the most unique part of a given transcript, making the detection of closely related genes possible [25]. A major advantage of oligonucleotide arrays is that they require no handling and tracking of cDNA resources [23]. Furthermore, the use of synthetic reagents in the manufacturing of oligonucleotide arrays minimizes variation among arrays, thus ensuring a high degree of reproducibility between microarray experiments.

Sample preparation is similar for cDNA and oligonucleotide microarrays. In both cases, mRNA is extracted, reverse transcribed to cDNA, labelled, and hybridized to probes on the surface of the array [25]. Two fluorescent dyes allow cDNA from two treatment populations to be labelled with different colors. When mixed and hybridized to the same array, the differentially labelled cDNA results in competitive binding of the target to the probes on the array. After hybridization and washing, the slide is imaged using a scanner and fluorescence measurements are made separately for each dye at each spot on the array. This dual labelling enables the ratio of transcript levels for each gene on the array to be determined [3,25]. Specialized software and data management tools are then used for data extraction and analysis.

### Application of microarrays in plant physiology

### Case study 1: developmental analysis of gene expression — cotton mutants with altered fibre characteristics and elongation of maize roots at low soil water potential

Cotton fibres are single-celled trichomes that differentiate from the epidermal layer of developing cotton ovules. They synchronously undergo a phase of rapid cell expansion, then a phase of secondary cell wall deposition, and finally maturation [30,33]. Cotton fibres are, as a result, unique in being one of the longest and most rapidly expanding plant cells, and thus can serve as a valuable experimental system to study fundamental processes in plant biology [10]. Research has shown that the main elongation period of cotton fibre starts on the day of anthesis and is completed roughly 21 days later, overlapping with the onset of secondary cell wall synthesis. Many genes are known to be transcriptionally regulated during this period of development [17]. However, progress in characterizing genes specifically involved in cell elongation has been slow. Such study has been hindered by the

Copyright © 2003 John Wiley & Sons, Ltd. 

long time required to regenerate transgenic cotton lines [34] and the lack of suitable transient assay systems [11].

One way to circumvent these difficulties and thus gain insights into the molecular mechanisms that control cell elongation in developing cotton fibres is through the use of mutants that have altered fibre elongation kinetics. As described in a poster by Arpat and Wilkins [2] from the University of California (Davis, CA), the *Pilose* (*Gossypium hirsutum* L.) mutant in cotton is a dominant, simply inherited nuclear mutation that is phenotypically characterized by mature fibres that are much shorter than those of conventional varieties. Fibre length measurements over the course of development revealed that the difference in mature fibre length was due primarily to altered growth rate, and not to early termination or late initiation of the elongation period in the *Pilose* mutant.

With this as their model system, the authors used cDNA microarrays to dissect the underlying molecular mechanisms that were responsible for the decreased growth observed in *Pilose* fibre. Cotton cDNA microarrays contained 4875 cDNAs representing a unigene set of expressed sequence tags (ESTs) sequenced from isolated fibres 7–10 days post-anthesis. Each probe was replicated twice on each slide. Cotton, *Arabidopsis* and non-plant sequences were also spotted in replicate to provide a measure of non-specific hybridization facilitated by poly-T tails of the cDNA probes.

One of the more important findings of this research was that four different targets representing $\alpha$-expansins on the cotton cDNA array had five-fold suppression in expression for fibres from the *Pilose* mutant. Expansins are cell wall proteins that, through pH-dependent processes, modify the mechanical properties of cell walls, leading to cell wall loosening and turgor-driven cell extension [5]. The data indicate that as a part of decreased cell growth rate, $\alpha$-expansins are among the most strongly suppressed gene groups in elongating *Pilose* fibres. Previous research has shown that two $\alpha$-expansin genes (GhExp1 and GhExp2) give rise to transcripts that are specific to developing cotton ovules [9] and it is suggested that GhExp1 may play an important role in cell wall extension during development of cotton fibres. Arpat and Wilkins [2] use data from their microarrays to question, however, whether the suppression of $\alpha$-expansins is the primary and/or only determinant of decreased fibre length in the *Pilose* mutant. For example, profilin, an accessory protein of actin filaments, was also suppressed in *Pilose*. Profilins have complex biochemical activities and multiple modes of action on the actin cytoskeleton. This observation supports the view that the *Pilose* phenotype involves multiple mechanisms, including both expansins and cytoskeleton dynamics. Future studies will be required to explore these ideas. Plans are already under way to develop full time-series expression profiles for both control and *Pilose* mutants in order to better understand the mechanisms that underlie the altered phenotype.

The research philosophy outlined by Arpat and Wilkins [2] for understanding the molecular mechanisms of fibre elongation in cotton was similar to that described by Sharp *et al.* [28] from the University of Missouri (Columbia, MO), who presented an overview of a project in which microarrays will be used to characterize gene expression for root elongation in maize (*Zea mays* L.). This work follows a long history by this author to understand the mechanisms of root growth at low soil water potentials [27]. Building upon this foundation, the broad aim of this project is to now develop an understanding of the molecular mechanisms that facilitate water and mineral acquisition by roots, to elucidate the role that roots play in adaptation to water-deficit conditions, and to transfer this knowledge to crop improvement through biotechnology. The project has recently been initiated through funding provided by the National Science Foundation, and seeks to identify the genes and biochemical networks that control the mechanisms of root growth and root-to-shoot communication under drought. Sharp and colleagues recently showed that maintenance of cell elongation in the apical region of maize primary roots at low water potentials was associated with an increase in transcript levels for several expansin genes [36], as well as an increase in expansin activity and extractable expansin protein [35]. Sharp's group has also demonstrated that accumulation of abscisic acid (ABA) is required for maintenance of maize primary root growth under water deficits [29,31]. These findings will be explored in the future with the use of microarrays to more fully characterize transcript profiles in elongating and non-elongating regions of roots under water deficit in sensitive

Copyright © 2003 John Wiley & Sons, Ltd. 

and tolerant maize lines, an ABA-deficient mutant, and near-isogenic lines differing in ABA accumulation. Such expression profiles will be combined with changes in protein profiles, especially cell wall proteins, in different regions of the root to identify factors associated with root growth maintenance and tolerance to water deficit. Knowledge gained will lead to novel approaches for improving drought tolerance in maize through genetic and metabolic engineering of root functions. A Plant Root Genomics Consortium has been formed and a website is available listing project goals, consortium members and tentative research strategies (**http://rootgenomics.missouri.edu/index.htm**).

### Case study 2: identification and characterization of genes responding to temperature stress in Chinese cabbage using cDNA microarrays

One of the widespread applications of microarrays in plant physiology is the molecular characterization of gene expression during exposure of plants to stress. This application is typified by a project carried out by Lee *et al.* [16] from the Chungnam National University (Daejon, Korea), in which the temperature response of Chinese cabbage (*Brassica rapa* var. *pekinensis*) was assessed using a cDNA microarray with 2688 leaf ESTs specific to this species. Treatments included a light chilling stress (5 °C) and a heat shock (32 °C) treatment under uniform light and moisture conditions. Leaf samples were collected from treatment and control plants at 1, 2, 4, 8 and 12 h following initiation of each treatment and mRNA was extracted, labelled and hybridized to the array.

Hierarchical clustering of genes which showed at least an eight-fold change with chilling stress revealed four distinct classes of response. Upregulated genes (115 in total) could be divided into two groups; in one, transcript levels initially increased and then decreased with time, and in the other, transcript levels increased continuously throughout the treatment period. Among these were genes previously demonstrated to be involved in cold stress, circadian rhythms, membrane lipid transfer, drought resistance, lipid metabolism and various membrane transporters and transcription factors. Downregulated genes (112 in total) could also be divided into two groups; in one, transcript levels gradually decreased and then increased, and in the other, transcript levels exhibited a continual decrease with time. Downregulated genes included those involved in photosynthesis (Rubisco activase), cell and cell wall growth, cellular signalling and many genes of unknown function.

In the case of heat shock, hierarchical clustering of genes with at least a four-fold change in expression also revealed four distinct classes of response. Upregulated genes (60 in total) could be divided into two groups, in the first of which transcript levels were maximum during the first hour of treatment and then afterwards decreased — most of these genes were heat shock proteins (e.g. heat shock protein 90). Transcript abundance was also high after 2 h of treatment and many of this second group were defence-related genes (e.g. cysteine-rich antifungal protein 4 precursor (AFP4), jasmonate-inducible protein). Downregulated transcripts (30 in total) included genes associated with growth inhibition (e.g. auxin-repressed protein) and cell signalling (e.g. protein kinase).

Many of these expression patterns were confirmed by independent Northern blot analyses and, according to Lee *et al.* [16], the function of individual genes up- and downregulated by light chilling and heat-shock treatments will be pursued in greater detail during future studies. These studies raise the possibility of identifying novel genes involved in heat and cold temperature response, and the transient nature of these temporal patterns of response. In this regard, the strongly up- and downregulated proteins of unknown function are of particular interest. In addition, these analyses could yield insights into the coordinated, multigenic responses to temperature stress, and could possibly lead to the discovery of 'master regulators' [4] that could be future targets of genetic engineering.

### Case study 3: microarray analysis of transcript abundance in barley under conditions of water deficit

Transcript profiling can provide useful information for the identification of candidate genes underlying quantitative trait loci (QTLs) for yield and other agronomic traits in crop plants grown under a range of conditions. In particular, there is widespread interest in using DNA microarrays to monitor gene expression profiles for crops exposed to limited soil water availability. Previous studies have shown that stress-inducible genes can be readily detected

Copyright © 2003 John Wiley & Sons, Ltd. 

in plants exposed to rapid dehydration [21,26]. Seki *et al.* [26] used microarrays containing 1300 full-length cDNAs to identify 44 drought-inducible genes in *Arabidopsis* following a 2 h dehydration, whereas Ozturk *et al.* [21] recently used cDNA microarrays to identify large-scale changes in transcript abundance for barley (*Hordeum vulgare* L.) exposed to rapid (i.e. 6–10 h) drought shock treatments.

Although information regarding expression profiles in response to the development of abrupt and severe water stress is useful, studies that impose slower, more realistic rates of dehydration are needed, as are studies that compare the resulting gene expression profiles to those from plants exposed to drought-shock treatments. In an attempt to meet this need, the presentation of Talamé and colleagues [32] from the University of Bologna, Italy was particularly relevant, as these authors described a study in which gene expression patterns were compared between several water stress treatments in young barley plants. A 'water-shock' experiment was conducted in which plants were grown in sand up to the four-leaf stage, at which time water was withheld by removing plants from the sand and leaving them to dehydrate for 6 h. A second, more conventional 'water-stress' experiment involved growing potted plants in a greenhouse up to the four-leaf stage and then slowly withholding water for 11 days until the moisture content of the soil mix declined to ca. 40% of maximum water-holding capacity. mRNA from tissues of treated and control plants was extracted, labelled and hybridized to microarrays containing 1463 DNA elements derived from barley cDNA libraries [21]. Changes in signal intensity between control and treated plants exceeding a 2.2-fold difference in two replicated experiments were considered significant.

Microarray analysis with mRNA extracted from water-shock and control tissues largely confirmed the changes in the expression level of transcripts, as reported earlier by Ozturk *et al.* [21]. Nearly 15% of all transcripts were either up- or downregulated under conditions of rapid drought stress. Transcripts that showed significant upregulation included jasmonate-responsive, metallothionein-like, late-embryogenesis-abundant (LEA) and ABA-responsive proteins. In the second experiment, water-stressed plants showed significant changes in transcript abundance after the seventh day of water deficit, when the relative water content was ca. 90–93%. The greatest change in expression was observed after 11 days of treatment. As in the water-shock experiment, expression profiles were analysed by cluster analysis and it was shown that the majority of treatment effects were due to transcript changes in genes whose putative function involved protein synthesis and turnover early in the experiment, followed by later induction of known stress-responsive transcripts.

Because it is difficult to monitor and control changes in the water status of plants under field conditions, experiments investigating the molecular response to drought are usually carried out under controlled conditions. The experimental protocols that are routinely applied to mimic drought conditions differ greatly in terms of the dynamics and/or intensity of the water stress treatments applied. Therefore, as nicely shown in the research of Talamé *et al.* [32], it becomes important to verify the correspondence of changes in expression profiles occurring under different experimental conditions mimicking drought field conditions and to verify to what extent such changes are relevant for the final performance of the crop. The comparative analysis of the two sets of microarray data showed that the expression of a relatively large set of transcripts was differently regulated under water-shock or water-stress treatments. In general, the water-stress experiment showed a lower number of transcripts being affected, as well as a lower intensity ratio between stressed and control treatments. Although in some cases similar classes of transcripts were up- or downregulated under conditions of water-shock and water-stress experiments, the overall correlation between transcript expression profiles in the two experiments was low. These results indicate that changes in expression vary considerably according to the dynamics of the water stress treatment. This suggests that only a portion of genes obtained with intense and rapid dehydration may predict the changes occurring when water stress develops in a slower fashion, more likely to occur under field conditions.

### Case study 4: profiling expression of aluminium-tolerance genes in wheat with microarrays

Aluminium is a major factor limiting crop productivity in acid soils, causing a substantial reduction

Copyright © 2003 John Wiley & Sons, Ltd.

in yield for many plants [7]. The primary effect of aluminium toxicity is inhibition of root elongation. Different species or crop cultivars may respond to aluminium differently and there is the potential that these responses have a genetic and/or molecular basis [14]. Some plants detoxify aluminium in the rhizosphere by releasing organic acids that chelate aluminium prior to root uptake, while other plants detoxify aluminium internally by forming complexes with organic acids [20]. Unfortunately, for the vast majority of plants, the precise mechanism by which plants tolerate exposure to metals in general, and aluminium specifically, remains largely unknown.

In the hopes of better understanding the molecular mechanisms associated with aluminium tolerance in wheat (*Triticum aestivum* L.), the poster of Guo *et al.* [8] from Oklahoma State University (Stillwater, OK) described how these authors used cDNA microarrays to assess differences in gene expression for tolerant and susceptible cultivars exposed to aluminium. Seedlings of the cultivar Chisholm (Al-sensitive) and its near-isogenic line Chisholm-T (Al-tolerant) were grown in hydroponic culture. The aluminium tolerance of Chisholm-T was derived from the Al-tolerant cultivar Atlas 66. Three day-old seedlings were exposed to a high but sub-lethal, concentration of aluminium (10 mg/l). Twenty-four hours later, the seedlings were transferred to deionized water and rinsed. Aluminium-stressed and control roots were stained for 15 min with haematoxylin. High-intensity staining indicates accumulation of a high level of aluminium in roots. Light microscopy of roots 24 h after exposure to aluminium clearly showed that the roots of Atlas 66 and Chisholm-T were lightly stained, whereas the roots of the Al-sensitive cultivar Chisholm were very dark.

Microarrays used in this study were created by using suppression subtractive hybridization to identify transcripts that differed between cultivars after exposure to aluminium [6]. This technique has been developed for the generation of subtracted cDNA libraries and combined normalization and subtraction into a single procedure. Sequence tags from 1628 differentially expressed cDNA clones were spotted on the array. The arrays were hybridized with cDNA from roots of Chisholm-T and Chisholm at 6 h, 1 day, 3 days and 7 days after aluminium stress.

Ninety-six differentially expressed genes (64 unigenes) were identified during at least one of the four sampling periods. These genes were clustered into six groups according to their expression patterns; three groups were generally upregulated and three were generally downregulated. Genes that showed strong differential expression between cultivars were sequenced, and putative functions were determined by homology searches. Genes that were upregulated in the tolerant wheat cultivar in at least one time point included those coding for lipid transfer protein, acetylglutamate kinase-like protein, jasmonate-induced protein, $\beta$-glucosidase, cellulose synthase peroxidase, and two disease resistance response proteins. Although all analyses are exploratory, the results of this research should facilitate understanding the pathways of expression and regulation of wheat genes under aluminium stress and provide insight into the genetic control of aluminium tolerance in wheat and other agricultural cereals.

### Discussion

There is little doubt that plant biology is in the midst of being revolutionized through the increasing availability of microarrays and their use in global gene expression studies [22]. As illustrated in this review, gene expression arrays are already being used to assess qualitative differences between plants exposed to temperature, drought and aluminium toxicity. Microarrays are also providing insights into spatial and temporal changes in gene expression associated with fibre and root elongation in cotton and maize, respectively. It will only be a matter of time before microarrays are used in a variety of physiologically relevant studies. These tools will, of course, be useful in studying the action of individual genes. It is hoped, however, that gene expression arrays will enable the actions of these individual genes to be studied and interpreted in a broader context. It should be possible to couple physiological measurements with gene expression profiles, thus illuminating the function of genes, biochemical pathways and cellular processes of interest to plant physiologists. Studies such as these would lay the groundwork for mapping regulatory networks and depicting linkages among gene products, biochemistry and

Copyright © 2003 John Wiley & Sons, Ltd.

whole-plant physiology. This could, in turn, facilitate predictive modelling of cellular processes and, with time, genome-based modelling of plant growth and physiology in response to a range of cultural, edaphic and climatic perturbations [37].

Despite their impressive and growing acceptance in the biological sciences, the utility of microarrays in plant physiology is still being explored. As such, there is ample opportunity and, indeed, a need for technical improvements and further development of this technology. John Quackenbush from The Institute for Genomic Research (Rockville, MD) emphasized in his presentation [13] that sequencing of the *Arabidopsis thaliana* genome largely set the stage for global studies of gene expression in plants. Comprehensive DNA microarrays are now available that represent the entire gene content of the nuclear, chloroplast and mitochondrial genomes. These arrays have been used to survey patterns of gene expression in a variety of physiologically-relevant situations, including analysis of plant response to oxidative stress and metabolic changes induced by elevated carbon dioxide concentrations. However, Quackenbush also emphasized that in order to interpret results from these experiments in a biologically meaningful manner, plant scientists should recognize that: (a) reasonable results demand intelligent, carefully designed experiments; (b) as they tend to be ubiquitously expressed, known genes are only part of the story, and hypothetical and unknown genes may be the key to interpreting plant responses; and (c) the challenge in using microarrays will be in analysing the data in a manner that yields new information. This latter point is particularly noteworthy in that long lists of genes with altered expression can easily be generated in microarray experiments, but such lists provide few clues as to which of these changes are important in establishing a given biochemical or physiological response. For example, a given stimulus might potentially lead to changes in the mRNA levels of hundreds of genes (see studies highlighted in this article) and the temptation will be to look within these patterns of response for genes that conform to existing ideas about how the system might work [25]. Analytical methods such as the hierarchical clustering of genes will be required to move past this temptation. Furthermore, the combination of transcript profiling with experimental treatments of single gene knock-out and knock-in transgenic plants will also help to deconvolute the complex response pathways of plants.

Finally, microarrays are being used in innovative ways. Kirst and colleagues [12] from the Forest Biotechnology Group at North Carolina State University (Raleigh, NC) described in their presentation a technique of treating microarray expression data as quantitative traits that could then be mapped in a segregating pedigree. These authors used a mixed-model ANOVA that simultaneously normalized the data by expression level, and associated gene expression levels with the presence of molecular markers from one or the other parent. Transcripts that showed differential expression were then assessed for association with quantitative traits, as is typically done with putatively neutral molecular markers. Transcripts with differential expression could also be mapped with traditional methods by assaying segregating polymorphisms in the coding regions, thus distinguishing between *cis*- and *trans*-acting factors affecting gene expression levels. As a proof of principle, Kirst *et al.* [12] used this approach to map a candidate gene associated with wood density in *Eucalyptus*. The identified gene was highly conserved among species, and was apparently involved in the regulation of cellular osmotic pressure. Thus, the gene could be one associated with the expansion of xylem cells during differentiation. This particular gene has not been previously identified as being involved in determining wood density, so this finding is novel. The approach has great potential to speed the discovery of genes controlling quantitative traits with physiological relevance, particularly in recalcitrant perennial species like trees, for which experimentation and functional genomics approaches are limited.

### Conclusion

Although difficulties in experimental design, sample collection, data analysis and the technical shortcomings of existing microarrays should not be underestimated, one should also not forget that, as with any new technology, time will be required before microarrays begin to yield their full wealth of information. As this technology matures, plant physiologists should consider how best to use these and other tools, technologies and resources that are becoming common in a post-genomic world. The

Copyright © 2003 John Wiley & Sons, Ltd. 

case studies presented here illustrate how microarrays are being used today, and provide a glimpse as to how they might be improved and used in the plant sciences tomorrow. The investigators featured in this review and others are breaking new ground. Their work with microarrays is evidence that plant scientists are taking the first steps toward gaining improved physiological understanding through the use of genomic approaches.

### Acknowledgements

Special thanks to the many investigators featured in this article. These individuals openly shared data, ideas and future plans, and ultimately consented to the representation of their research in this report. Oak Ridge National Laboratory is managed by UT-Battelle, LLC, for the US Department of Energy under contract No. DE-AC05-00OR22725. Abstracts for all posters and presentations referenced in this paper can be obtained through the searchable database at the Plant and Animal Genome Conference website (**http://www.intl-pag.org**).

### References

1. Aharoni A, Vorst O. 2002. DNA microarrays for functional plant genomics. *Plant Mol Biol* **48**: 99–118.
2. Arpat AB, Wilkins TA. 2003. Gene expression analysis of cotton Pilose mutant with altered fibre characteristics. Plant and Animal Genome Conference XI, 11–15 January 2003, San Diego, CA, p. 267.
3. Brown PO, Botstein D. 1999. Exploring the new world of the genome with DNA microarrays. *Nature Genet* **21**(suppl): 33–37.
4. Chapin FS III. 1991. Integrated responses of plants to stress: a centralized system of physiological responses. *Bioscience* **41**: 29–36.
5. Cosgrove DJ, Li LC, Cho HT, *et al.* 2002. The growing world of expansins. *Plant Cell Physiol* **43**: 1436–1444.
6. Diatchenko L, Lau YFC, Campbell AP, *et al.* 1996. Suppression subtractive hybridization: a method for generating differentially regulated or tissue-specific cDNA probes and libraries. *Proc Natl Acad Sci USA* **93**: 6025–6030.
7. Drummond RD, Guimaraes CT, Felix J, *et al.* 2001. Prospecting for genes involved in aluminium tolerance. *Genet Mol Biol* **24**: 221–230.
8. Guo P-G, Bai G-H, Carver B. 2003. Profiling expression of aluminium-tolerance genes in wheat through microarrays. Plant and Animal Genome Conference XI, 11–15 January 2003, San Diego, CA, p. 265.
9. Harmer SE, Orford SJ, Timmis JN. 2002. Characterization of six $\alpha$-expansin genes in *Gossypium hirsutum* (upland cotton). *Mol Genet Genomics* **268**: 1–9.
10. Kim HJ, Triplett BA. 2001. Cotton fibre growth *in planta* and *in vitro*. Models for plant cell elongation and cell wall biogenesis. *Plant Physiol* **127**: 1361–1366.
11. Kim HJ, Williams MY, Triplett BA. 2002. A novel expression assay system for fibre-specific promoters in developing cotton fibres. *Plant Mol Biol Rep* **20**: 7–18.
12. Kirst M, Myburg AA, Sederoff RR. 2003. Genetical genomics of *Eucalyptus*: combining expression profiling and genetic segregation analysis. Plant and Animal Genome Conference XI, 11–15 January 2003, San Diego, CA, p. 36.
13. Klapa M, Kim H, Linford L, *et al.* 2003. Global gene expression analysis in *Arabidopsis thaliana*. Plant and Animal Genome Conference XI, 11–15 January 2003, San Diego, CA, p. 72.
14. Kochian LV, Pence NS, Letham DLD, *et al.* 2002. Mechanisms of metal resistance in plants: aluminium and heavy metals. *Plant Soil* **247**: 109–119.
15. Kuo WP, Jenssen T-K, Butte AJ, *et al.* 2002. Analysis of matched mRNA measurements from two different microarray technologies. *Bioinformatics* **18**: 405–412.
16. Lee D-S, Song H, Kim Y, *et al.* 2003. Identification and characterization of genes responding to temperature stresses in Chinese cabbage using cDNA microarray. Plant and Animal Genome Conference XI, 11–15 January 2003, San Diego, CA, p. 268.
17. Li CH, Zhu YQ, Meng YL, *et al.* 2002. Isolation of genes preferentially expressed in cotton fibres by cDNA filter arrays and RT–PCR. *Plant Sci* **163**: 1113–1120.
18. Lipshutz RJ, Fodor SPA, Gingeras TR, *et al.* 2002. High density synthetic oligonucleotide arrays. *Nature Genet* **21**: 20–24.
19. Lockhart DJ, Winzeler EA. 2000. Genomics, gene expression and DNA arrays. *Nature* **405**: 827–836.
20. Ma JF, Ryan PR, Delhaize E. 2001. Aluminium tolerance in plants and the complexing role of organic acids. *Trends Plant Sci* **6**: 273–278.
21. Ozturk ZN, Talamè V, Deyholos M, *et al.* 2002. Monitoring large-scale changes in transcript abundance in drought- and salt-stressed barley. *Plant Mol Biol* **48**: 551–573.
22. Schaffer R, Landgraf J, Perez-Amador M, *et al.* 2000. Monitoring genome-wide expression in plants. *Curr Opin Biotechnol* **11**: 162–167.
23. Schena M, Heller RA, Theriault TP, *et al.* 1998. Microarrays: biotechnology's discovery platform for functional genomics. *Trends Biotechnol* **16**: 301–306.
24. Schena M, Shalon D, Davis RW, *et al.* 1995. Quantitative monitoring of gene expression patterns with a complementary DNA microarray. *Science* **270**: 467–470.
25. Schulze A, Downward J. 2001. Navigating gene expression using microarrays — a technology review. *Nature Cell Biol* **3**: E190–E195.
26. Seki M, Narusaka M, Abe H, *et al.* 2001. Monitoring the expression pattern of 1300 *Arabidopsis* genes under drought and cold stresses by using full-length cDNA microarray. *Plant Cell* **13**: 61–72.
27. Sharp RE, Silk WK, Hsiao TC. 1988. Growth of the maize primary root at low water potentials. 1. Spatial distribution of expansive growth. *Plant Physiol* **87**: 50–57.
28. Sharp RE, Bohnert HJ, Davis GL, *et al.* 2003. Root growth maintenance during water deficits: Physiology to functional genomics. Plant and Animal Genome Conference XI, 11–15 January 2003, San Diego, CA, p. 5.

Copyright © 2003 John Wiley & Sons, Ltd. 

29. Sharp RE, Wu Y, Voetberg GS, *et al*. 1994. Confirmation that abscisic acid accumulation is required for maize primary root elongation at low water potentials. *J Exp Bot* **45**: 1743–1751.
30. Smart LB, Vojdani F, Maeshima M, *et al*. 1998. Genes involved in osmoregulation during turgor-driven cell expansion of developing cotton fibres are differentially regulated. *Plant Physiol* **116**: 1539–1549.
31. Spollen WG, LeNoble ME, Samuels TD, *et al*. 2000. ABA accumulation maintains primary root elongation at low water potentials by restricting ethylene production. *Plant Physiol* **122**: 967–976.
32. Talamè V, Ozturk N, Bohnert HJ, *et al*. 2003. Microarray analysis of transcript abundance in barley under conditions of water deficit. Plant and Animal Genome Conference XI, 11–15 January 2003, San Diego, CA, p. 15.
33. Wilkins TA, Jernstedt JA. 1999. Molecular genetics of developing cotton fibres. In *Cotton Fibres*, Basra AM (ed.). Hawthorne Press: New York; 231–267.
34. Wilkins TA, Rajasekaran K, Anderson DM. 2000. Cotton biotechnology. *CRC Crit Rev Plant Sci* **15**: 511–550.
35. Wu YJ, Sharp RE, Durachko DM, *et al*. 1996. Growth maintenance of the maize primary root at low water potentials involves increases in cell-wall extension properties, expansin activity, and wall susceptibility to expansins. *Plant Physiol* **111**: 765–772.
36. Wu YJ, Thorne ET, Sharp RE, *et al*. 2001. Modification of expansin transcript levels in the maize primary root at low water potentials. *Plant Physiol* **126**: 1471–1479.
37. Wullschleger SD, Tuskan GA, DiFazio SP. 2002. Genomics and the tree physiologist. *Tree Physiol* **22**: 1273–1276.

Copyright © 2003 John Wiley & Sons, Ltd.
